# Supplementary material for: Empirical methods for controlling false positives and estimating confidence in ChIP-Seq peaks
Source: BMC Bioinformatics. 2008 Dec 5;9:523. doi: 10.1186/1471-2105-9-523 (PMC2628906; doi:10.1186/1471-2105-9-523)
Supplement: Additional File 1 — A variety of html documents from the USeq web site detailing the available applications, their best usage, output file type descriptions, command line menus, etc. [file 1471-2105-9-523-S1.zip › USeqUserGuides/JavaDoc.zip!/JavaDoc/edu/utah/seq/parsers/package-summary.html]

edu.utah.seq.parsers


|  |  |  |
| --- | --- | --- |
| **Overview** \| **Package** \| Class \| **Use** \| **Tree** \| **Deprecated** \| **Index** \| **Help** | |  |
| **PREV PACKAGE**   **NEXT PACKAGE** | **FRAMES**    **NO FRAMES**     **All Classes** |  |


---

## Package edu.utah.seq.parsers

Parsers for converting sequencing data into binary PointData, xxx.bar.zip files.

**See:**
  
          **Description**

| **Class Summary** | |
| --- | --- |
| **BarParser** | Class for reading and writing xxx.bar files. |
| **ElandExportLine** |  |
| **ElandMultiParser** | Parses an Eland xxx.eland\_multi.txt file tabulating hits to each fasta entry. |
| **ElandParser** | Parses an Eland extended \_export.txt or \_sorted.txt file into point data, split by chromosome and strand. |
| **ElandSequenceParser** | Parses an Eland Extended xxx.export.txt or xxx.sorted.txt for sequencing information. |
| **JohnsonToTag** |  |
| **Tag2Point** | Splits a tag file (chr start stop ... |

## Package edu.utah.seq.parsers Description

Parsers for converting sequencing data into binary PointData, xxx.bar.zip files.

---


|  |  |  |
| --- | --- | --- |
| **Overview** \| **Package** \| Class \| **Use** \| **Tree** \| **Deprecated** \| **Index** \| **Help** | |  |
| **PREV PACKAGE**   **NEXT PACKAGE** | **FRAMES**    **NO FRAMES**     **All Classes** |  |


---
